# Comparative Genomics within and across Bilaterians Illuminates the Evolutionary History of ALK and LTK Proto-Oncogene Origination and Diversification

**DOI:** 10.1093/gbe/evaa228

**Published:** 2020-11-16

**Authors:** Alex Dornburg, Zheng Wang, Junrui Wang, Elizabeth S Mo, Francesc López-Giráldez, Jeffrey P Townsend

**Affiliations:** 1 Department of Bioinformatics and Genomics, University of North Carolina Charlotte; 2 Department of Ecology and Evolutionary Biology, Yale University, New Haven; 3 Department of Biostatistics, Yale School of Public Health, New Haven, Connecticut; 4 Crop Sciences, Chinese Academy of Agricultural Sciences, Beijing, China; 5 Yale Combined Program in the Biological and Biomedical Sciences, Yale School of Medicine, Yale University, New Haven; 6 Department of Genetics, Yale Center for Genome Analysis (YCGA), Yale University, West Haven; 7 Program in Microbiology, Yale University, New Haven

**Keywords:** cancer evolution, vertebrates, genome duplication, phylogenomics, functional divergence, gene evolution

## Abstract

Comparative genomic analyses have enormous potential for identifying key genes central to human health phenotypes, including those that promote cancers. In particular, the successful development of novel therapeutics using model species requires phylogenetic analyses to determine molecular homology. Accordingly, we investigate the evolutionary histories of anaplastic lymphoma kinase (ALK)—which can underlie tumorigenesis in neuroblastoma, nonsmall cell lung cancer, and anaplastic large-cell lymphoma—its close relative leukocyte tyrosine kinase (LTK) and their candidate ligands. Homology of ligands identified in model organisms to those functioning in humans remains unclear. Therefore, we searched for homologs of the human genes across metazoan genomes, finding that the candidate ligands Jeb and Hen-1 were restricted to nonvertebrate species. In contrast, the ligand augmentor (AUG) was only identified in vertebrates. We found two *ALK*-like and four *AUG*-like protein-coding genes in lamprey. Of these six genes, only one *ALK*-like and two *AUG*-like genes exhibited early embryonic expression that parallels model mammal systems. Two copies of *AUG* are present in nearly all jawed vertebrates. Our phylogenetic analysis strongly supports the presence of previously unrecognized functional convergences of ALK and LTK between actinopterygians and sarcopterygians—despite contemporaneous, highly conserved synteny of *ALK* and *LTK*. These findings provide critical guidance regarding the propriety of fish and mammal models with regard to model organism-based investigation of these medically important genes. In sum, our results provide the phylogenetic context necessary for effective investigations of the functional roles and biology of these critically important receptors.

**Significance**

Model organisms have the potential to provide vital research breakthroughs that revolutionize human biology and medicine. However, inferences from model organisms often fail to translate to humans because of evolved differences in gene function and interaction. Such evolved differences currently stymie translational research on the cancer driver gene *ALK*, its sister *LTK*, and the genes they interact with. Our comparative analysis revealed their evolutionary history, demonstrating that *LTK* and *ALK* have switched and subsequently duplicated gene partners between invertebrates and vertebrates, and that these genes have exchanged functions between humans and fish model species. These results illuminate the model organisms with which future research will effectively translate to human cancer biology—specifically for guiding the development of novel therapeutics.

## Introduction

A diversity of nonhuman model species distributed across the Tree of Life has been essential to investigations of the biology of key genes responsible for trait evolution and human health phenotypes. Correspondingly, comparative investigations of the genes that prevent or promote the origin and spread of cancer have been fruitful for understanding human cancer biology (O’Hagan et al. 2005; Paoloni and Khanna 2008; Stoletov and Klemke 2008; Rowell et al. 2011; Kocher and Piwnica-Worms 2013). However, the challenge to such investigations is that gene function and regulation evolves. Underlying molecular divergences can become obscured by convergences in phenotype that can confound our ability to infer the homology of receptors and their interacting partners between species. Accordingly, accurate homology predictions have the potential to accelerate the development of novel therapeutics. Therefore, estimating the evolutionary history of oncogenes provides a critical reference for translation of fundamental findings in model organisms (Lemke 2015; Reshetnyak et al. 2015; Gudernova et al. 2017; Mo et al. 2017).

This problem of distinguishing homology from convergence has grown particularly acute within anaplastic lymphoma kinase (ALK) and leukocyte tyrosine kinase (*LTK*), two well-known receptor tyrosine kinase (RTK) proto-oncogenes whose roles in oncogenesis and potential as therapeutic targets have been increasingly investigated (Fujioka et al. 2006; Marzec et al. 2007; Soda et al. 2007; Chiarle et al. 2008; Bresler et al. 2011; Roll and Reuther 2012; Wellstein 2012; Hallberg and Palmer 2013; Holla et al. 2017; Lin et al. 2017). In particular, structural conservation of ALK between invertebrate models as diverse as fruit flies, nematodes, and humans, model organisms has been central to illuminating the biology of oncogenic alterations such as mutation type prevalences in cancer tissues (Ogawa et al. 2011), overexpression triggering abnormal activation of ALK (De Brouwer et al. 2010; Zhu et al. 2012), and ligand-dependent mutations (Mosse et al. 2008). However, disentangling potentially complex patterns of divergent and convergent evolution in ALK and LTK requires investigating three core aspects of evolutionary history, each establishing the extent to which model systems provide functional parallels of humans.

The first aspect of evolutionary history with translational relevance regards the homology of *ALK* and *LTK*. A gene that by a simple BLAST search appears to be *ALK* or *LTK* in one model organism could in fact be the other with a very divergent regulatory apparatus and functional repertoire. Second, key domains in ALK and LTK have been gained and lost and may have undergone convergent as well as divergent evolution. ALK and LTK have a tripartite structure including an intracellular kinase domain (KD), a single transmembrane domain, and an extracellular domain (ECD; Inoue and Thomas 2000; Englund et al. 2003; Bilsland et al. 2008; Weiss et al. 2012). Within the ECD, a low-density lipoprotein receptor class A (LDLa) repeat and two protein tyrosine phosphatase Mu (MAM) domains are also conserved, the latter playing a role in RTK homo-dimerization, which leads to rapid activation of the KDs across metazoans (Cismasiu et al. 2004). Knowing the history of gain, loss, and sequence evolution of these domains is essential to knowing functional parallels between model systems and humans. Third, three ligands of Alk have been identified: Jelly belly (Jeb) in *Drosophila melanogaster*, Hesitation behavior-1 (Hen-1) in *Caenorhabitis elegans* (Ishihara et al. 2002; Lee et al. 2003; Rohrbough and Broadie 2010), and Augmentor (FAM150 or AUG-α in *Homo sapiens*; Guan et al. 2015; Reshetnyak et al. 2015). Understanding the identity and functional interactions of these ligands with their cognate receptors has been argued to be vital to the development of inhibitors and other small-molecule pharmaceuticals (Grande et al. 2011; Slavish et al. 2011; Tartari et al. 2011; Gambacorti-Passerini 2016; Fadeev et al. 2018), yet the homology of these ligands identified in diverse organisms has not been established (Guan et al. 2015).

In this study, we performed an exhaustive search for homologs of vertebrate *ALK*, *LTK*, and augmentor (*AUG*) against genomes of organisms that include all major vertebrate lineages, additional chordates, hemichordates, and protostomes. Across these genomes, we identified genes homologous to those known to encode ALK ligands, providing evidence for the origins of AUG ligands as a vertebrate-specific innovation. We further reconstructed ancestral sequences of vertebrate AUGs and performed phylogenetic analysis of the evolution of vertebrate *ALK*s, *LTK*s, and *AUG*s to reveal the history of major events in their evolution, including the gains and losses of genes and the evolution of functional domains. Using the gene phylogenies obtained, we further identified amino acids that likely play essential roles in the functional divergence between gene paralogs. By determining the origins and evolution of the proto-oncogenic tyrosine kinases ALK and LTK and their ligands across the history of Metazoans, our results provide the necessary foundation for effective, phylogenetically informed investigations of the functional roles of these genes.

## Results

### The Evolutionary History of *ALK*, *LTK*, *JEB*, *HEN-1*, and *AUG* Homologs

BLAST searches (including BlastP and TBlastN) revealed a diversity of sequences that are potentially homologous to sequences of ALKs and associated ligands from model and nonmodel organisms across the genomes available at Ensembl (Kersey et al. 2017) and NCBI (NCBI Resource Coordinators 2017; supplementary table S1, Supplementary Material online). ALKs identified in nematode and fruit fly genomes exhibited sufficient conservation of sequence for homologous alignment in the GR and KDs. One homolog of vertebrate ALK (NCBI XP_032804141) was found in the sea lamprey (Smith et al. 2013, 2018b) and Japanese lamprey genomes (Mehta et al. 2013), that included identifiable MAM2, LDLa, and tyrosine KDs. In addition, a sequence with potential ALK similarity that was annotated as LTK was found (NCBI XP_032818754). This annotated LTK protein exhibited 65% sequence similarity with the predicted lamprey ALK protein along with conservation of a glycine-rich tyrosine KD. However, no MAM and LDLa domains were present in the annotated LTK protein. BlastP searches using this protein yielded hits on ALK and LTK proteins in birds and mammals; with the highest scoring hit against the human genome being the ALK (Ki-1) receptor, followed by ALK, then LTK. Consequently, sequence similarity alone was not sufficient to assign this gene to either ALK or LTK. Functional or structural domains of ALK, LTK, and ALK-like proteins were predicted with sequence comparison and by InterPro (Mitchell et al. 2019). We find orthologs of *ALK* are present in almost all vertebrate genomes as well as most protostomes. However, *ALK* appears to have been lost in the early diverging chordate lineage *Ciona*, as well as in hagfish—the sister lineage to lamprey. In contrast, we detected ALK-like and Jeb-like proteins within the genome of *lancelets*.

**Fig. 1:**
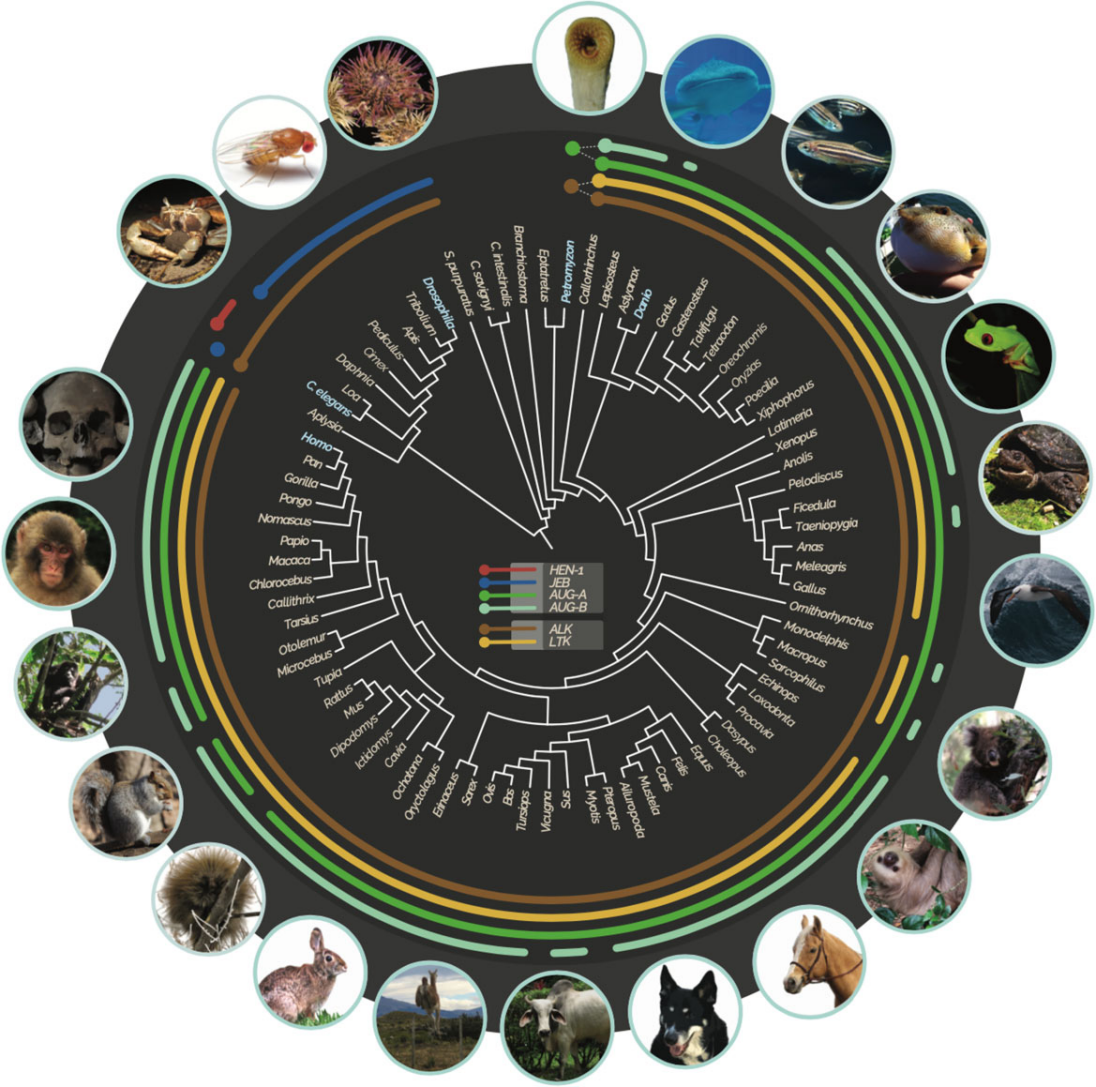
Phylogenetic distribution of *HEN-1* (red), *JEB* (blue), *ALK* (brown), *LTK* (yellow), *AUG-α* (green), and *AUG-β* (teal) orthologs in metazoan genomes (arcs span taxa with each gene; light blue taxon names correspond to model organisms discussed in the text). Identification of homologous genes was confirmed with phylogenetic analyses, and gene accession numbers are provided (supplementary table S1, Supplementary Material online; Photo credits Dan Warren [koala], Katerina Zapfe [squirrel], Bronwyn Williams [urchin], Matt Bertone [*Drosophila*], Lynn Ketchum [zebrafish: creative commons, cropped from the original], and Alex Dornburg [all others]).

**Fig. 2:**
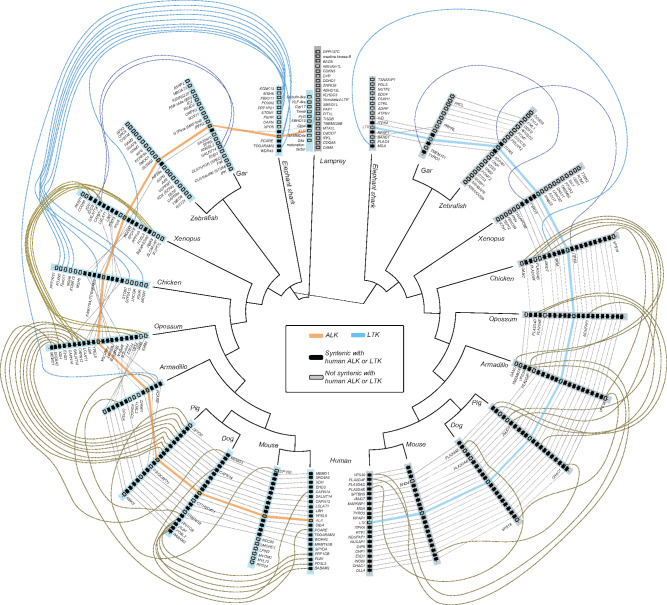
Synteny (dotted lines and curves) of up to ten genes (syntenic with human ALK, black rectangles; nonsyntenic with human ALK, light gray rectangles) that are annotated as present in genomes on either side of *ALK* (light blue bars; orange trace) and *LTK* (dark gray bars; blue trace) across major clades of vertebrates.

In a parallel to our finding of *ALK* and a candidate LTK homolog in the lamprey genomes, multiple AUG-like proteins (XP_032817706, XP_032809021, XP_032826340, XP_032810391) were also found in the sea lamprey and Japanese lamprey genomes. In contrast, no orthologs of *Jeb* or *Hen-1* were found in any vertebrates, with *Hen-1* restricted entirely to the nematodes *C. elegans* and *Loa*. *Jeb* was present in most protostomes: our identification of Jeb-like proteins in *Aplysia* and *Strongylocentrotus*, respectively, extends the presence of this ligand to mollusca and represents the first identification of this protein in a deuterostome. However, we found no evidence of *Jeb* in other chordate lineages, indicating that Jeb may have been lost prior to the diversification of vertebrates (fig. 1). As lampreys are members of the earliest-diverging lineage of living vertebrates, our finding of an *ALK*, a possible *LTK*, and multiple *AUG* genes is suggestive of two potential scenarios. One is that *ALK*, *LTK*, and two copies of *AUG* arose at the dawn of vertebrates. A second possibility is that a duplication of the *ALK*–*AUG* ligand–receptor gene pair gave rise to *LTK* and a gene for a second AUG ligand after the divergence of the lamprey lineage from the vertebrate ancestor and prior to the diversification of jawed vertebrates (Dehal and Boore 2005; Smith et al. 2018b; fig. 1). The latter hypothesis is supported by conserved synteny: genes adjacent to *ALK* and *LTK* in humans are found adjacent to *ALK* and *LTK* within lineages as divergent as *Anolis*, elephant sharks, and gar, with evidence for conserved synteny between lamprey and other jawed vertebrates restricted to genes surrounding *ALK* (fig. 2), thereby suggesting other lamprey genes to be lineage-specific paralogs.

**Fig. 3:**
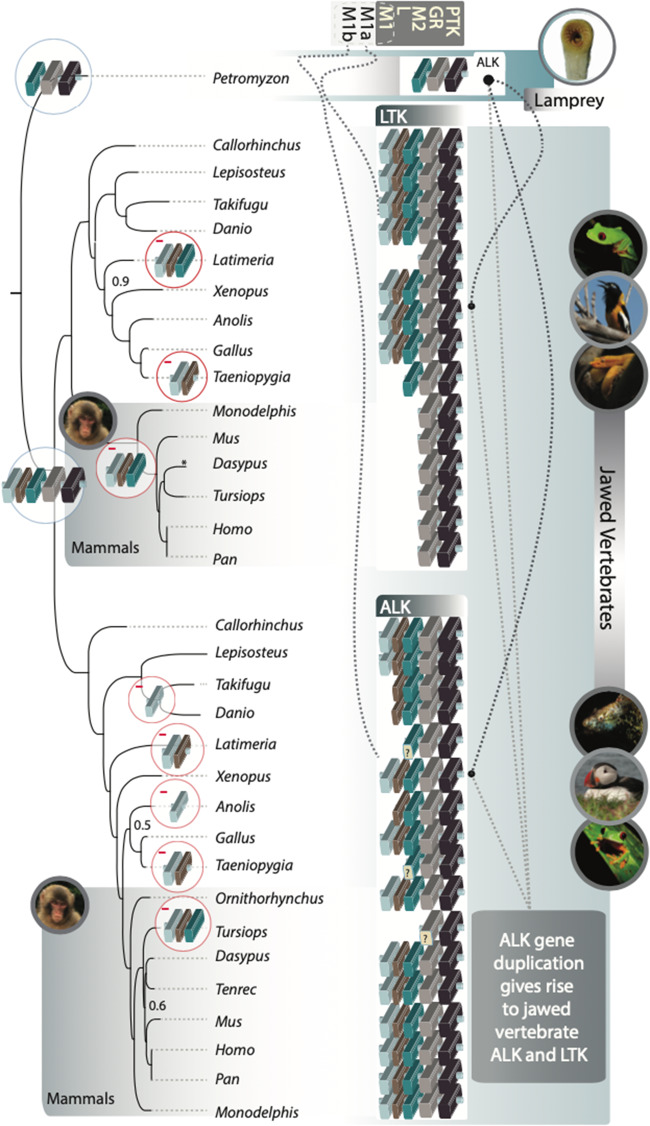
Phylogeny of vertebrate ALKs and LTKs. ALK in Lamprey (*Petromyzon*) features only GR and PTK domains (sand and onyx blocks). M_2_, L, and possibly M_1_ domains (teal, light brown, and light gray blocks) are duplicated in jawed vertebrates, giving rise to *ALK* and *LTK* (black dotted lines from simple *ALK*, indicated with gray lines from inset text). Dashed lines from MAM domains indicate the presence of M_1a_ and M_1b_ in ALK and LTK, respectively. Some domains in some lineages exhibited low levels of sequence similarity to domains observed in other vertebrates (“?”s on GR block on the lineage to *Tursiops*, and M2 on the lineage to both *Latimeria* and *Taeniopygia*). Domain losses (−) are indicated at internodes where they were reconstructed to occur (e.g., loss of M1, L, and M2 in LTK in mammals—highlighted with a gold gradient, and loss of M1, L, and M2 in *Tursiops* in ALK). Unlabeled internodes all exhibited strong statistical support (Bayesian posterior probability >0.98), labels on internodes indicate other BPP values. Photos: A.D.

Phylogenetic analysis of vertebrate ALK and LTK provided further strong support for a history in which the duplication of *ALK* gave rise to *LTK* prior to the most recent common ancestor (MRCA) of jawed vertebrates. The origin of *LTK* mapped to the MRCA of chondrichthyans and Osteichthyes (sarcopterygians + actinopterygians; fig. 1). Synteny of some genes neighboring *ALK* and *LTK* was conserved between and within major clades of vertebrates (fig. 2). Moreover, there was strong support for the reciprocal monophyly of jawed-vertebrate ALK and LTK (Bayesian posterior probability [BPP] = 1.0; fig. 3) and strong support for a clade comprising lamprey ALK and jawed-vertebrate ALK + LTK that excluded the annotated lamprey LTK protein (supplementary fig. S1, Supplementary Material online). Comparisons of the evolutionary history of ALK and LTK demonstrate divergent rates of molecular evolution as well as divergent patterns of domain acquisitions and losses (fig. 3). For example, mammals exhibit a signature of decelerated evolution of nonsynonymous substitution in *ALK*, a deceleration that contrasts with a significantly faster rate of molecular divergence of mammal *LTK*s (fig. 3; supplementary tables S4 and S6, Supplementary Material online). We found that both ALK (Iss = 1.08, Iss.c = 0.83, *P *<* *0.01) and LTK (Iss = 1.21, Iss.c = 0.83, *P *<* *0.01) have experienced severe amino acid substitution saturation (Xia and Lemey 2009; DAMBE; Xia 2018) and exhibit a sharp decline of phylogenetic informativeness (PhyInformR; Dornburg et al. 2016) within mammals (supplementary fig. S6, Supplementary Material online). Rates of evolution of ALK and LTK between “fish” (ray-finned fishes, sharks, and Coelacanth) and mammals contrasted: mammal LTK exhibited significant saturation (Iss = 1.07, Iss.c = 0.84, *P *<* *0.01) relative to “fish” (Iss = 0.67, Iss.c = 0.84); and ALK for “fish” exhibited significant saturation (Iss = 1.07, Iss.c = 0.84, *P *<* *0.01) relative to mammals (Iss = 0.65, Iss.c = 0.84). We found evidence for functional divergence in different sites between ALK and LTK, with six sites (745-Lys, 760-Leu, 767-Lys, 795-Ile, 808-Asn, and 863-Asn positions of human ALK) identified as important to the evolution of differential function of ALK and LTK (*P *<* *0.05; supplementary fig. S3, Supplementary Material online). These sites were all located between the MAM2 domain and the GR region, with the exception of 863-Asn, located in the GlyR domain. Functional divergence analyses further predicted 11 amino acids in human LTK to be of significant (*P *<* *0.01) importance in the differential function of LTK between mammals and nonmammals groups (98-Thr, 120-Leu, 152-Leu, 171-Gly, 200-Gly located before the GlyR domain and 216-Tyr, 226-Glu, 245-Arg, 261-Ala, 262-Pro, 267-Arg located within the GlyR domain), five of which are conserved across ALK and LTK in jawed vertebrates (supplementary fig. S4, Supplementary Material online).

### The Evolution of AUG in Vertebrates

**Fig. 4:**
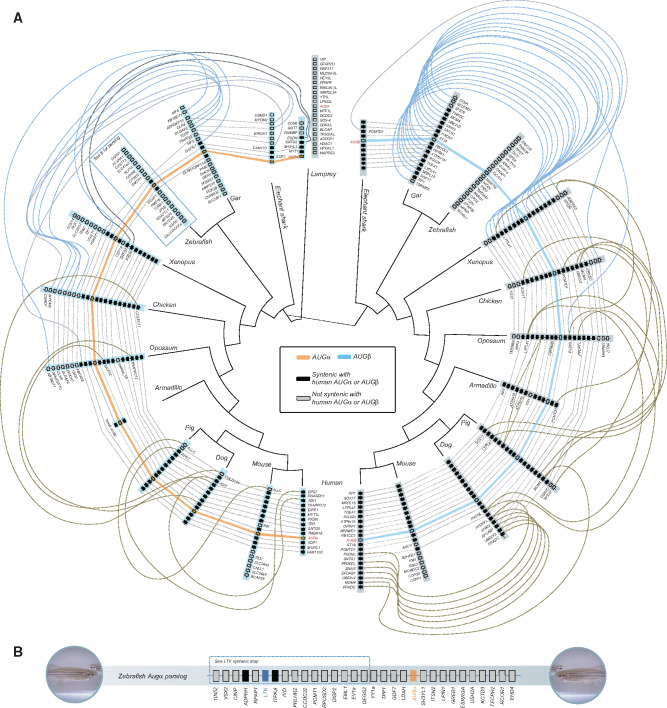
(*A*) Synteny (dotted lines or curves—blue, synteny to a nontetrapod; brown, synteny within tetrapods) of up to ten genes (syntenic with human *AUG-*α or *AUG-*β, black rectangles; not syntenic with human *AUG-*α or *AUG-*β, light gray rectangles) that are annotated in genomes as present on either side of *AUG-*α (light blue bars; orange trace) and *AUG-*β (dark gray bars; blue trace) across major clades of vertebrates. (*B*) In zebrafish, an *AUG-*α paralog is located within 15 genes of *LTK*.

**Fig. 5:**
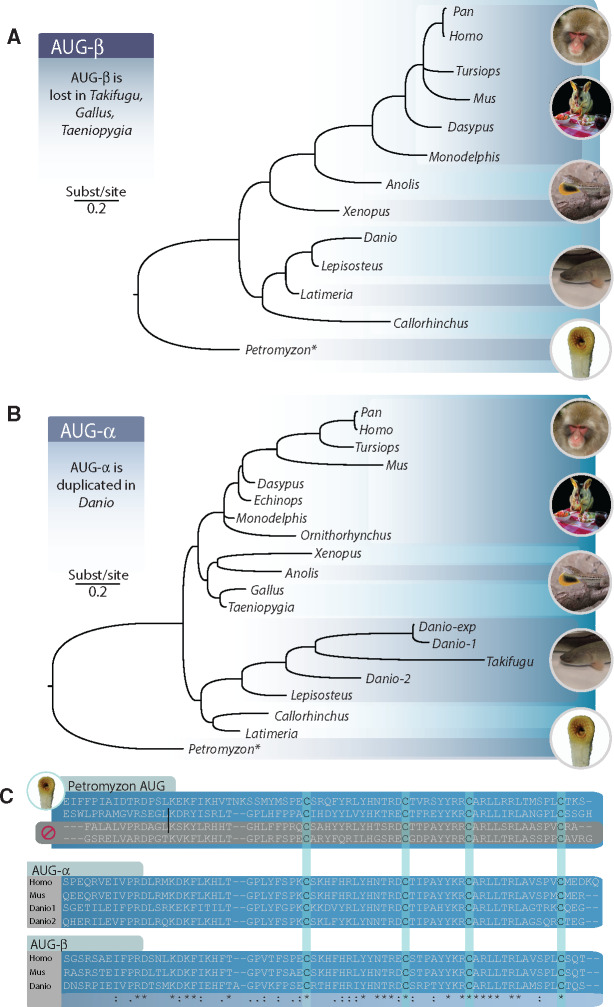
Phylogeny of vertebrate (*A*) AUG-β and (*B*) AUG-α, in which labels at branches indicate Bayesian posterior probabilities <0.98, illustrated with (*C*) an amino acid alignment of selected AUGs demonstrating sequence conservation between lamprey AUG and its homologs in mammals and zebrafish including key cysteine positions near the C-terminus (aquamarine columns). The lamprey alignment possessed unique sequence insertions (vertical lines) that are not shown here. An asterisk (*) indicates the Petromyzon sequence that is most similar to jawed-vertebrate AUG. Some lamprey AUG sequences have lost their signaling peptide (gray shading). Images: A.D. (Possum at dinner courtesy of Dan Warren).

Investigation of transcriptomes and sequenced genomes revealed that AUG is an innovation shared by all vertebrates. Using the annotated lamprey AUG sequence and the maximum-likelihood ancestral sequence for the MRCA of vertebrate AUG and lamprey AUG revealed no potential homologs in searches of any invertebrate deuterostome, protostome, or non-Metazoan genomes. Our reconstruction of the evolutionary history of *AUG* strongly supports its duplication into *AUG-α* and *AUG-β* prior to the MRCA of jawed vertebrates, suggesting that the other three *AUG* genes in lamprey are lamprey-specific paralogs (fig. 1 and supplementary fig. S5, Supplementary Material online). This hypothesis is further supported by the loss of signaling peptides in two of the lamprey *AUG* genes (XP_032826340, XP_032810391) and the absence of conserved amino-acid motifs shared by vertebrates in the N-terminus of AUG-α or AUG-β in XP_032809021. In parallel, conserved synteny supports duplication and divergence: genes adjacent to *AUG-α* or *AUG-β* in humans are also found adjacent to *AUG-α* or *AUG-β* across representative chondrichthyan, actinopterygian, and sarcopterygian lineages (fig. 4). Collectively these lineages span the MRCA of all jawed vertebrates (fig. 4) and—assuming complete and accurate genome annotation—provide evidence that absences of *AUG-α* (fig. 5*A*) or *AUG-β* (fig. 5*B*) are a consequence of heterogeneous lineage-specific losses. Further, sequence comparisons of jawed-vertebrate AUG-α and AUG-β reveal these ligands to share structural conservation with lamprey AUG: all encode four cysteines near the C-terminus (fig. 5*C*). Functional divergence analysis further identified three sites in human AUG-α (81-Glu, 91-Leu, and 146-Val) as being significant (*P *<* *0.01) to the differential function of the AUG paralogs.

Orthologs of AUG-α exhibited rapid evolution (high nonsynonymous substitution) across jawed vertebrates, suggesting a comparatively conserved function of AUG-β (table 1). This hypothesis is supported by a relative ratio test demonstrating significantly greater numbers of amino-acid substitutions in AUG-α than in AUG-β (*P *<* *0.05). Both AUG-α (Iss = 1.39; Iss.c = 0.76) and AUG-β (Iss = 1.45; Iss.c = 0.76) have experienced severe substitution saturation (DAMBE; Xia and Lemey 2009; Xia 2018) and by the sharp decline of phylogenetic informativeness (PhyInformR; Dornburg et al. 2016) of the protein sequence at timescales corresponding with the divergence of mammals (supplementary fig. S6, Supplementary Material online).

**Table 1:** Evolutionary Rates Estimated for ALK, LTK, AUG-α, and AUG-β Based on Pairwise Comparisons under the LPB Model in PAML for Mammals, “Fishes” (Ray-Finned Fishes, Sharks, and Coelacanths), and Other Nonmammalian Tetrapods

Category	Species	ALK	LTK	AUG-α	AUG-β
Mammals	*Homo* versus *Monodelphis*	0.1033	0.2439	0.2471	0.2002
	*Pan* versus *Monodelphis*	0.0994	0.2469	0.2524	0.1909
	*Dasypus* versus *Monodelphis*	0.0728	0.4182	0.3076	0.2153
	*Dasypus* versus *Homo*	0.0639	0.53	0.4664	0.2778
	*Dasypus* versus *Pan*	0.0632	0.5325	0.4814	0.2874
	*Mus* versus *Monodelphis*	0.0985	0.2392	0.2228	0.1339
	*Mus* versus *Homo*	0.0727	0.2725	0.4479	0.4256
	*Mus* versus *Pan*	0.0658	0.272	0.4612	0.4378
	*Mus* versus *Dasypus*	0.0574	0.4504	0.386	0.1527
Fishes	*Danio* versus *Callorhinchus*	0.1196	0.1718	0.5046	0.3557
	*Latimeria* versus *Callorhinchus*	0.1858	0.1496	0.4482	0.1616
	*Lepisosteus* versus *Callorhinchus*	0.3171	0.1389	0.5743	0.2639
	*Lepisosteus* versus *Latimeria*	0.1859	0.1251	0.176	0.2994
Others	*Anolis* versus *Taeniopygia*	0.1728	0.1633	0.3636	0.2057
	*Xenopus* versus *Taeniopygia*	0.1447	0.1554	0.4406	0.2052
	*Xenopus* versus *Anolis*	0.1475	0.1718	0.4283	0.1727

### Tissue-Specific Expression Profiles of ALK and AUG in Lamprey

Quantifying expression levels of *ALK* and *AUG* transcripts in lampreys identified similarities in expression that are consistent with their operation as a ligand–receptor pair and that align with function in humans. We performed PCR-based (RACE) strategies in muscle, brain, liver, and eye tissues from adult and ammocoete lampreys (supplementary table S2, Supplementary Material online) and analyzed RNA sequencing data from 76 experiments in eight transcriptomic projects of *Petromyzon marinus* in NCBI Sequence Read Archive (SRA). Short reads were aligned to the recently updated annotation of *P. marinus* genome from NCBI, and we quantified expression as raw read counts that mapped to the two *ALK*-like genes and four *AUG*-like genes (supplementary table S3, Supplementary Material online). For *ALK* and the annotated LTK gene, expression of the annotated *LTK* (XP_032818754) was detected in 67 out of the 76 experiments with an average 55 counts (maximum 483 counts), whereas *ALK* (XP_032804141) exhibited very low expression (1–12 counts) in 20 out of the 76 experiments as well as our RACE results. This suggests a restricted role for XP_032804141 in lamprey development. Expression of *AUG* was not detected from our lamprey specimens in either the ammocoete or the adult life stage. Among the four AUG-like genes, expression of XP_032826340 and XP_032810391—which have no predicted signal peptides—was either undetectable or very low in all 76 experiments, whereas AUG-XP_032817706 and AUG-XP_032809021 were, respectively, detected in 55 and 61 of the 76 experiments, always at high expression levels (supplementary fig. S7, Supplementary Material online). We found expression levels of the two *AUG* homologs—summed, as would be consistent with subfunctionalization—were correlated with expression of *ALK*, supporting the hypothesis that AUG and ALK are a ligand–receptor pair in lamprey (Pearson’s correlation coefficient *r*(84) = 0.59, *P *<* *10^−5^; supplementary table S3 and fig. S7, Supplementary Material online). These findings are consistent with the hypothesis that they were a ligand–receptor pair in the common ancestor of lamprey and jawed vertebrates. *AUG* transcripts were found in the neurula stage, olfactory tissues after exposure to copper, meiotic testes and brains, and samples with postinjury spinal cord and brain tissue (supplementary table S3, Supplementary Material online). No transcripts of *ALK*, *AUG*-XP_032817706, or *AUG*-XP_032809021 were detected in eight samples, including parasitic olfactory epithelium, adult olfactory epithelium, adult brain, parasitic larval brain, laval liver, parasitic liver, parasitic kidney, and parasitic liver samples (supplementary table S3, Supplementary Material online).

## Discussion

Here we have reported our discovery that the AUG ligands of ALK and LTK are a vertebrate innovation, and demonstrated that invertebrate genes *JEB* and *HEN-1* are not homologs of *AUG*. This lineage-specific evolution of ALK and LTK function across Metazoa likely underlies a diversity of hypotheses regarding how human functions and phenotypes of ALK homologs relate to the functions and phenotypes observed in protostome models such as nematodes and fruit flies (Ishihara et al. 2002; Hallberg and Palmer 2013, 2016; Reshetnyak et al. 2018). The split between *ALK*-bearing vertebrates and *ALK*-bearing protostomes corresponds not only to a divergence in receptor–ligand association but also to a greater functional divergence. For instance, ALK plays a central role in the visceral gut formation, growth, and neurogenesis in protostomes (Gouzi et al. 2011; Wolfstetter et al. 2017), and a role in neuronal proliferation, differentiation, and survival in vertebrates (Weiss et al. 2012; Yao et al. 2013). Similarly, we have demonstrated that after the genesis of *AUG*, an additional duplication likely occurred prior to the MRCA of jawed vertebrates, giving rise to the ligands AUG-α and AUG-β. This duplication of *AUG* coincided with a duplication of anaplastic lymphoma kinase (*ALK*) creating leukocyte tyrosine kinase (*LTK*).

The duplication of an ancestral *ALK* into *ALK* and *LTK* reveals striking functional similarities between these two lineages of tyrosine kinases. These functional similarities within ALK, within LTK, and between ALK and LTK encourage the use of a wide range of candidate vertebrate model species for investigation of the receptors, ligands, and their interactions. For instance, the LTKs of nontetrapods, including models such as zebrafish, exhibit a strong signature of shared domain structure with mammal ALK. The only notable difference is the sequence divergence between the N-termini of ALK and LTK within the first MAM domain. In addition, we also found evidence for the conservation of 11 key amino acids between nontetrapod and mammal LTK. The amino-acid identity of five of these 11 amino acids is—remarkably—also shared between nontetrapod LTK and mammal ALK. This conservation is encouraging and consistent with previous research emphasizing the importance of the nonhuman models in ALK tumorigenesis (Hallberg and Palmer 2013). Experimental research investigating the effects of induced point mutations in the ALK and LTK sequences at the sites identified here would be especially likely to reveal functionally divergent aspects of ALK and LTK signaling among humans and relevant model species.

Structurally, we found lamprey ALK is similar to mammalian LTK: both feature the PTK and GR domains and lack MAM1 and LDL domains. Our rapid-amplification-of-cDNA-ends experimental results and our analysis of public transcriptomic data mapping to lamprey *ALK* and *AUG* demonstrated that *ALK* and *AUG* are coexpressed postfertilization. Postfertilization expression and maximal expression during early embryonic development and after injury to nerve or brain tissues suggests a role of ALK and AUG in nerve and brain development. These results are consistent with expression of *ALK* in mice and humans that is also highest during embryonic development, quickly drops after birth, and is subsequently maintained at a low level (Iwahara et al. 1997; Vernersson et al. 2006). Together, our results and prior studies suggest that the functional roles of ALK and AUG may be conserved across vertebrates. Future research examining spatiotemporal changes in *ALK* and *AUG* expression between species—in particular in response to stressors—presents an exciting and potentially fruitful avenue toward an increasingly thorough understanding of the general role of these genes in humans and as well as their role in human cancers.

Our phylogenetic analyses of AUG-α revealed an accelerated evolutionary rate that is unexpected for proteins executing critical biological functions. Lower rates of sequence evolution are typically expected for proteins believed to have a collocalized dual specificity of interaction between genes. Biophysical binding data suggest AUG-α is a dual-specific ligand for both ALK and LTK (Reshetnyak et al. 2015). Our results demonstrate that this dual specificity has not constrained the evolution of AUG-α to a slower substitution rate within mammals. Instead, molecular rates of AUG-α exceed those estimated for AUG-β, which is a monospecific ligand of only LTK (Reshetnyak et al. 2015). The high substitution rates observed in mammal LTK, nontetrapod ALK, and jawed vertebrate AUG-α could indicate increased functional specificity and lower promiscuity of interaction in these genes. In contrast, we found AUG-β homologs to be more conserved than AUG-α homologs, a signature consistent with expectations of coevolution between signaling and receiving molecules (Goh et al. 2000; Laisney et al. 2010; Monte et al. 2018). In mammals, ALK is activated via both ligand-dependent (Reshetnyak et al. 2015; Takita 2017) and ligand-independent (Perez-Pinera et al. 2007; Deuel 2013) processes, implying multiple functions of ALK and high interaction specificity between ALK and its ligand(s). These lower substitution rates potentially indicate promiscuous interactions among AUG-β and its receptors. Biological relevance of these interactions has been indicated by research on both zebrafish development and human cancers (Guan et al. 2015; Reshetnyak et al. 2015; Mo et al. 2017). The differences in evolutionary rates between AUG-α and AUG-β, mammal LTK and nontetrapod ALK, and mammal ALK and nontetrapod LTK represent evolutionary trade-offs between functional specificity and the number of interactors a protein can achieve (Zhang and Yang 2015).

Rapidly evolving proteins have been shown to exhibit greater functional specificity—for example, higher tissue specificity or higher promoter methylation in mammals (Zhang and Yang 2015). If the different rates of evolution of these receptors and their ligands are the outcomes of evolutionary trade-offs, we might expect a higher complexity of the protein networks associated with mammal LTK and nontetrapod ALK, and expect more functional generality in networks associated with mammal ALK and nontetrapod LTK. Consequent hypotheses that mammal LTK and nontetrapod ALK regulate nerve development in conjunction with many highly specialized partner proteins, and that mammal ALK and nontetrapod LTK play broadly important and general roles in internal developmental signaling, warrant further molecular biological investigation. Additional collection of data on genome-wide or gene-specific spatial and temporal coexpression in vertebrates would provide additional insight into regulatory gene interaction networks, narrowing the scope of viable hypotheses for protein–protein interaction experiments as well as revealing evolutionary change in the structure and function of the ALK, LTK, and AUG signaling networks. Building on the evolutionary history we have here revealed, comparative analyses of interspecific protein interaction networks will reveal how these genes and gene domains are co-opted in tumorigenesis and cancer progression. Such insights will enable translational research toward interventions that successfully target the cellular function of ALK and LTK in human cancers.

## Materials and Methods

### Identification Homologs of ALK and Its Possible Ligand in Nonmodels

To investigate homologs of vertebrate ALKs, LTKs, and their associated ligands across model organisms spanning the protostomes and deuterostomes, amino-acid alignments of ALK homologs as well alignments of JEB, HEN-1, and AUGs from the genomes of zebrafish (*Danio rerio*), fruit fly (*D. melanogaster*), and nematode (*C. elegans*) were used to perform HMMER ortholog searches (Wheeler and Eddy 2013) against available genomes from genome databases in NCBI (www.ncbi.nlm.nih.gov) and Ensembl (www.ensembl.org; supplementary table S1, Supplementary Material online). For genomes whose annotation did not report homologs of these genes, additional BlastP and TBlastN searches were conducted with protein sequences derived from the three representative genomes listed above. Sequences with the mutually best matches between two sequences in genome pairs via BLAST search (Moreno-Hagelsieb and Latimer 2008) were subjected to further phylogenetic analyses to confirm their homology with annotated ALK, LTK, or ligand proteins. To investigate the evolution of ALK/LTK and associated AUG in vertebrates, sequences from model species that included zebrafish, frog (*Xenopus tropicalis*), chicken (*Gallus gallus*), zebra finch (*Taeniopygia guttata*), mouse (*Mus musculus*), and human (*H. sapiens*) were queried against nonmodel vertebrate genomes (supplementary table S1, Supplementary Material online) using HMMER and best-hit reciprocal-BLAST searches. To illuminate the origin of vertebrate ALK/LTK and AUG, special attention was devoted to thoroughly ascertain the presence of ALK and possible ligand(s) in the genomes of the jawless vertebrates (hagfish and lampreys), as these animals represent the earliest-diverging extant vertebrate lineage (Shimeld and Donoghue 2012). No sequence in the hagfish genome exhibited any similarity to vertebrate ALK, LTK, or AUG. BLAST searches of multiple ALK and AUG sequences from nontetrapod genomes recovered highly conserved regions between the two lamprey genomes indicating the presence of homologs of jawed-vertebrate ALK and AUG. To analyze synteny of ALK, LTK, and AUG orthologs among vertebrate representatives, predicted genes around targets were extracted from genome annotations of the sea lamprey, elephant shark, zebrafish, *Xenopus*, chicken, armadillo, pig, dog, mouse, and human genomes available from the UCSC genome browser, as well as from the NCBI genome browser for gar, and from the Ensemble genome browser for the opossum.

Searches for *ALK*, *LTK*, and paired ligands were additionally conducted against transcriptomes of sea lampreys (*P. marinus*), including 86 publicly available transcriptomes (supplementary table S3, Supplementary Material online). RNAs were additionally sampled from tissues of an ammocoete and an adult sea lamprey. The ammocoete lamprey was flash-frozen in liquid nitrogen before tissues of head, muscle, and viscera were dissected for RNA extraction. Tissues of the large adult lamprey were dissected from muscle, eyes, liver, brain, and heart. All tissues were preserved in RNA*later*, then maintained at −76 °C prior to RNA extraction. Total RNA was extracted from homogenized tissue with TRI REAGENT (Molecular Research Center). Messenger RNA was purified using Dynabeads oligo(dT) magnetic separation (Invitrogen). A cDNA library was generated using a SMARTer 5′/3′ RACE Kit (Takara cat no. 634860) as per the manufacturer’s instructions. First-strand cDNA synthesis was performed using 11 μl of RNA extract and 1 μl of 3′-CDS Primer A. Rapid amplification of cDNA ends (RACE) was also performed as per the manufacturer’s instructions with custom gene-specific primers (supplementary table S2, Supplementary Material online).

The genome sequence of the lamprey *P. marinus* was downloaded from Ensembl (Aken et al. 2017) and was used as the reference sequence for HISAT2 (Kim et al. 2015) to build the index and perform read alignment. Transcripts were assembled and gene-expression levels were quantified using StringTie (Pertea et al. 2015). Sequence read data totaled 323 Gb, and the largest single data set (based on paired-end sequencing with the Illumina HiSeq 4000 platform) amounted to 21.7 Gb. We used the most recently annotated *P. marinus* genome at NCBI as a reference for mapping reads, specifying the *HHEX* gene as a control (Sharman and Holland 1998). Due to its high efficiency, HISAT2 (Kim et al. 2015) was chosen to map the reads, which were subsequently extracted with SAMtools (Li et al. 2009) using parameter setting -F 4 to filter unmapped reads. All RNAseq data sets (supplementary table S3, Supplementary Material online) generated by previous studies (Libants et al. 2009; Chang et al. 2013; Ren et al. 2015; Bryant et al. 2016; Goetz et al. 2016; Ravi et al. 2016, 2019; Zhang et al. 2017; Herman et al. 2018; Smith et al. 2018a; Jones et al. 2019; Chung‐Davidson et al. 2020) and downloaded from NCBI. Reads were derived from samples of whole embryos 1- to 2.5-day postfertilization; neural crest, kidney, brain, liver, and olfactory tissue after 24-h exposure to 5, 10, and 30 g/ml of copper; and brains (whole brains without the olfactory lobes) and spinal cords (1 cm surrounding the lesion), harvested from 6 h to 12 weeks after injury of lamprey specimens (supplementary table S3, Supplementary Material online).

### Molecular Phylogeny

We obtained amino-acid sequences and nucleotide sequences of *ALK*/*LTK* and *AUG-α*/*AUG-β* genes from NCBI and Ensemble, respectively (supplementary table S1, Supplementary Material online). The amino-acid sequences of ALK and Jeb in *D. melanogaster*, and SCD-2 (homologous with ALK in *Drosophila*) and Hen-1 in *C. elegans*, were accessed for sequence comparison to lamprey ALK and AUG. We subsequently used lamprey sequences as outgroups for phylogenetic inference of ALK/LTK and AUG evolution in jawed vertebrates. Amino-acid sequences were aligned using MAFFT in Saté-II (Liu et al. 2012; Katoh and Standley 2013), whereas nucleotide sequences were aligned based on amino-acid sequences provided by the TranslatorX online server (Abascal et al. 2010). We inferred phylogenies of ALK and AUG using Markov chain Monte Carlo (MCMC) methods implemented in MrBayes 3.2 (Ronquist et al. 2012). Our Bayesian phylogenetic analyses were executed for 10,000,000 generations, sampling every 1,000 generations with four chains. We set lamprey ALK and AUG as outgroups for each analysis and discarded 2,500 (25%) of the 10,000 trees as burn-in. We assessed convergence of the chains by quantifying potential scale reduction factors (PSF = 1.0). We visually compared computed log likelihoods across chains to confirm stationarity. For nucleotide sequences, the GTR + I + Γ substitution model was specified. For amino acid sequences, a mixed model of amino acid substitution was specified that allowed each model of amino acid substitution to contribute in relation to its posterior probability. We deemed branches that exhibited a posterior probability (PP) higher than 0.98 to be strongly supported (figs. 2 and 3; supplementary fig. S1, Supplementary Material online). We conducted analyses on both the aligned amino acid and nucleotide sequences separately.

### Ancestral Reconstruction

Using phylogeny reconstructed for ALK and AUG homologs in vertebrates, ancestral sequence reconstructions were performed using both likelihood and Bayesian approaches as implemented in PAML 4 (Yang 2007). We used codeml to conduct codon-based ancestral sequence reconstruction of the common ancestor of mammals as well as all vertebrates. Reconstructed sequences of ALK and AUG for the common ancestor of lampreys and jawed vertebrates were also used to search against nonvertebrate genomes for possible homologs. Ancestral states of MAM domains in vertebrate genomes were estimated using maximum-likelihood (ML) criteria in BayesTraits (Pagel 1999; Pagel and Meade 2004). We coded the presence or absence of MAM domains for ALK and LTK, and used the multiState method of discrete character evolution to reconstruct gains or losses of MAM domains (supplementary fig. S2, Supplementary Material online).

### Selection Tests

To test for positive selection along specific branches in vertebrate ALK/LTK and AUG evolution, we used branch models implemented in PAML (Yang 1997, 2007), in which the ratio of nonsynonymous to synonymous substitution (*ω*) was allowed to vary among branches in the phylogeny (supplementary tables S4 and S5, Supplementary Material online). The ratio *ω* was estimated for the branch of interest (the “foreground” branch) and the rest of the tree (the “background”) in the phylogeny reconstructed from a multiple sequence alignment. To evaluate whether there was a statistically significant difference between the branch model and the null model, a likelihood-ratio test (LRT) was applied. To search for positively selected sites, site models permitting ω to vary among sites were used. We set NSsites to equal 0, 1, 2, 7, and 8, then conducted likelihood-ratio tests between pairs of the models to identify the best fitting model comparing M_1a_ (nearly neutral) against M_2a_ (positive selection), and M_7_ (beta) against M_8_ (beta and *ω*), each with two degrees of freedom. A Bayes-Empirical Bayes analysis was performed to identify the sites evolving under significant positive selection (Yang et al. 2005). Clade-model C was fit to the data to evaluate whether *ω* differed between ALK and LTK and between AUG-α and AUG-β in mammals (Bielawski and Yang 2004; Yang et al. 2005). The improved model M_2a_rel_ was used as the null model for the likelihood-ratio test on clade-model C results (Weadick and Chang 2012).

The degree of saturation of substitutions for ALK, LTK, and AUG proteins was assessed by DAMBE (Xia and Lemey 2009) and by inspection of phylogenetic informativeness profiles (Townsend 2007) visualized with the R package PhyInformR (Dornburg et al. 2016). To estimate site rates, we used HyPhy (Cummings 2004) within the PhyDesign web interface (López-Giráldez and Townsend 2011). Profiles of phylogenetic informativeness were depicted along a relative ultrametric guide topology generated in BEAST v. 2.4.7 (Drummond et al. 2012) with a prior root height of 1.0. As the depths of divergence examined exhibited some evidence of saturation with regard to substitutions, we compared their maximum-likelihood rates of sequence evolution using a likelihood-ratio test conducted in Hyphy (Cummings 2004), enabling meaningful comparisons of relative-rate differences between AUG paralogs.

### Functional Divergence Analysis

We used DIVERGE 3.0 (Gu et al. 2013) to test for functional divergence of the gene pairs. DIVERGE tests for site-specific shifts in evolutionary rates after gene duplication or speciation. The coefficient of divergence (*θ_D_*) was calculated to test against a null hypothesis of no functional divergence between ALK and LTK, between mammal ALK and fish ALK, between mammal LTK and fish LTK, and between AUG-α and AUG-β. We employed the default posterior probability cutoff of 0.5 for detection of site-specific shifted evolutionary rates (Gu et al. 2013). Amino acids with significant (*P *<* *0.05) roles in functional divergence between gene paralogs were predicted.

## Supplementary Material

Supplementary data are available at *Genome Biology and Evolution* online.

## Supplementary Material

evaa228_Supplementary_Data
