# Supplementary material for: Comparative Genomics within and across Bilaterians Illuminates the Evolutionary History of ALK and LTK Proto-Oncogene Origination and Diversification
Source: Genome Biol Evol. 2020 Nov 16;13(1):evaa228. doi: 10.1093/gbe/evaa228 (PMC7851593; doi:10.1093/gbe/evaa228)
Supplement: evaa228_Supplementary_Data [file evaa228_supplementary_data.pdf]

Supplemental Materials

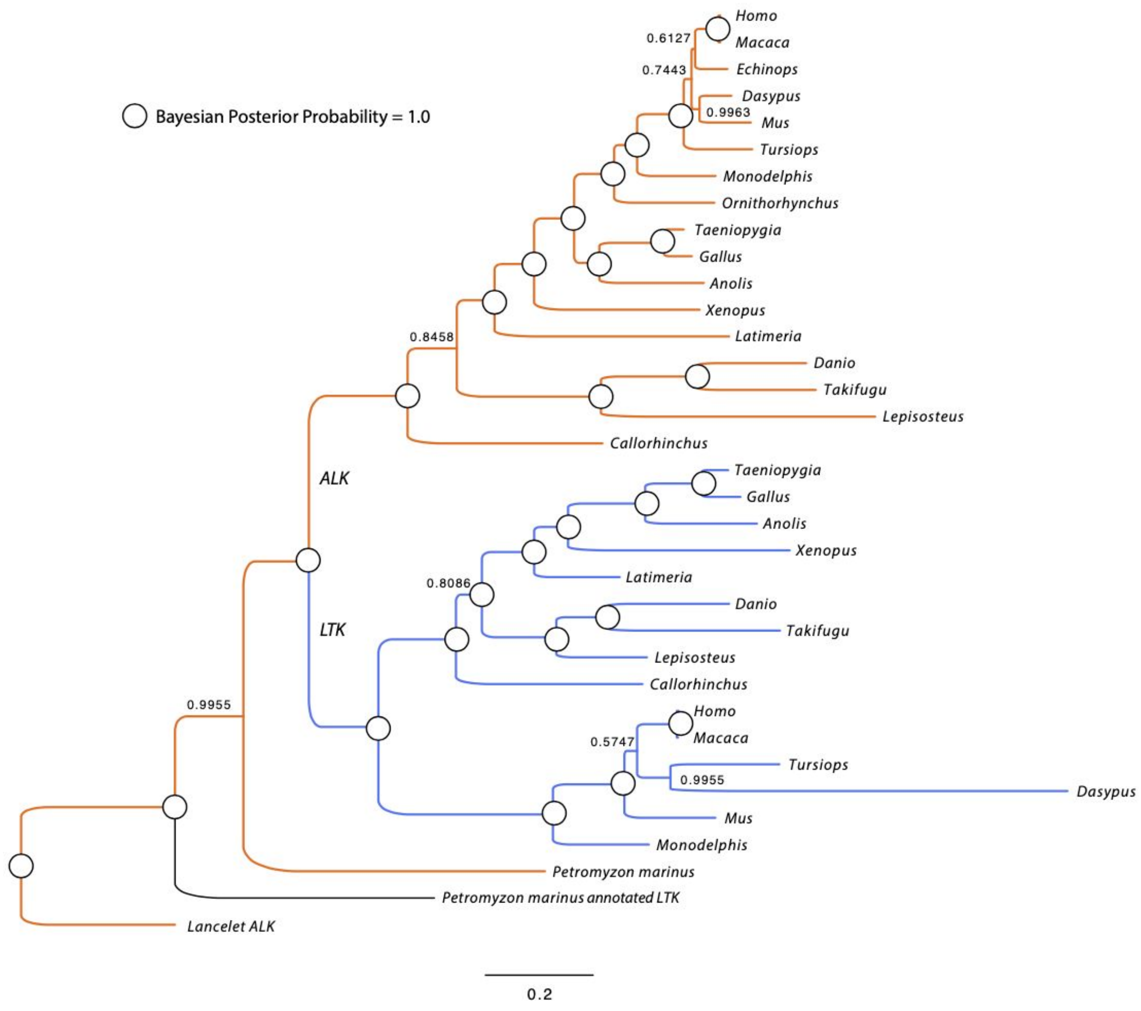

Figure S1. Phylogeny of vertebrate ALK and LTK, rooted with Lancelet ALK. The phylogeny was inferred from a joint amino-acid alignment of ALK and LTK using `MrBayes` (93, 94). Branches with strong support (PP = 1.0) are indicated by circles. Orange lines indicate the lineages homologous to human ALK, blue indicates the lineages homologous to human LTK.

	M1 Absence	M1b	M2	M1a	M2 Absence	M1b	M2	M1a
Node A	0.92	0.05	0.01	0.02	0.92	0.05	0.01	0.02
Node B	0.00	0.00	0.00	1.00	0.00	0.00	1.00	0.00
Node C	0.00	1.00	0.00	0.00	0.00	0.00	1.00	0.00
Node D	0.00	0.41	0.00	0.59	0.00	0.00	1.00	0.00
Root	0.00	0.47	0.00	0.53	0.00	0.00	1.00	0.00

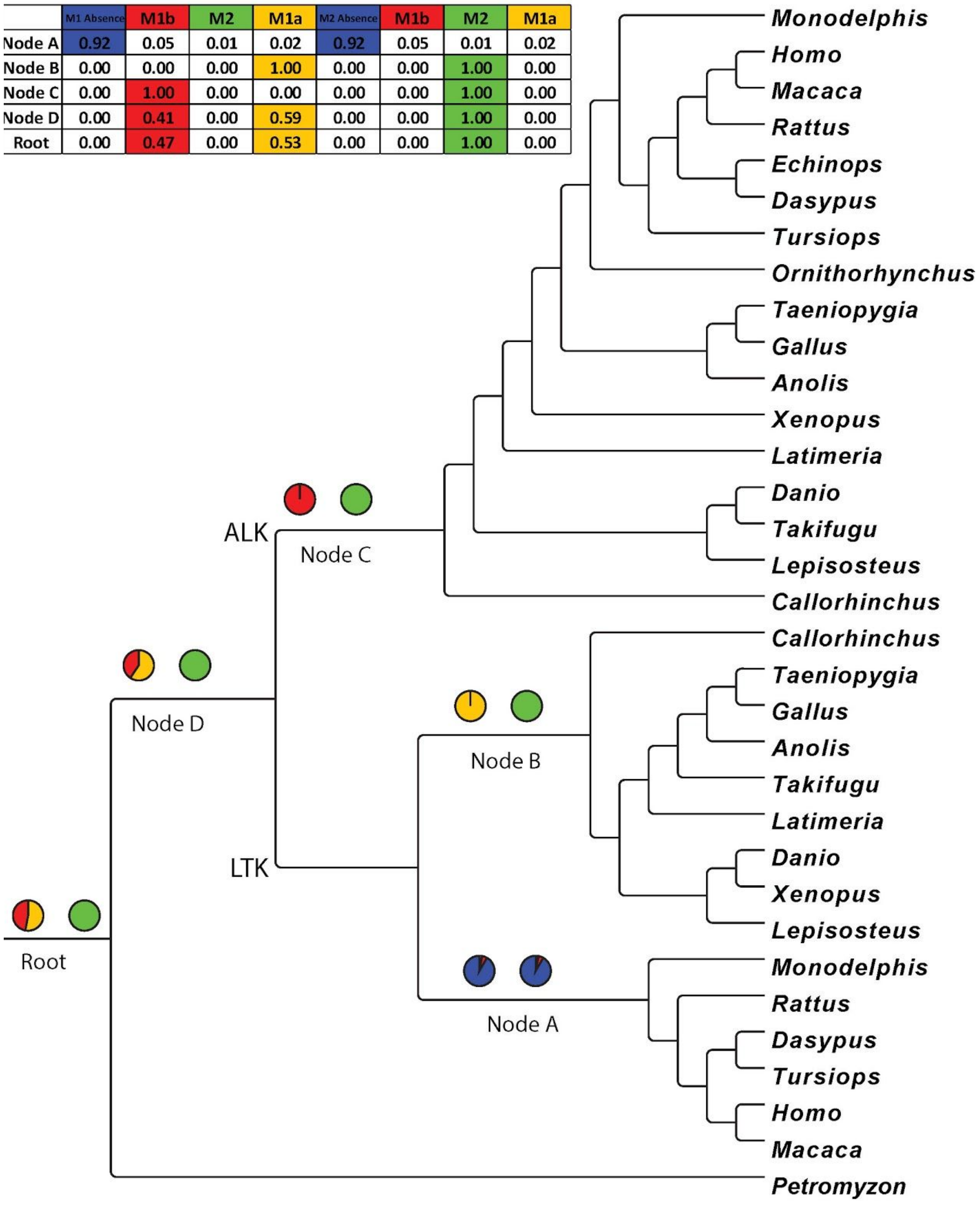

Figure S2. Estimated ancestral states of MAM domains for vertebrate ALK and LTK using maximum likelihood (ML) in `BayesTraits` (82, 83). MAM domains are classified as MAM1a (M1a, red), MAM1b (M1b, yellow), and MAM2 (M2, green). Absence was coded in blue.

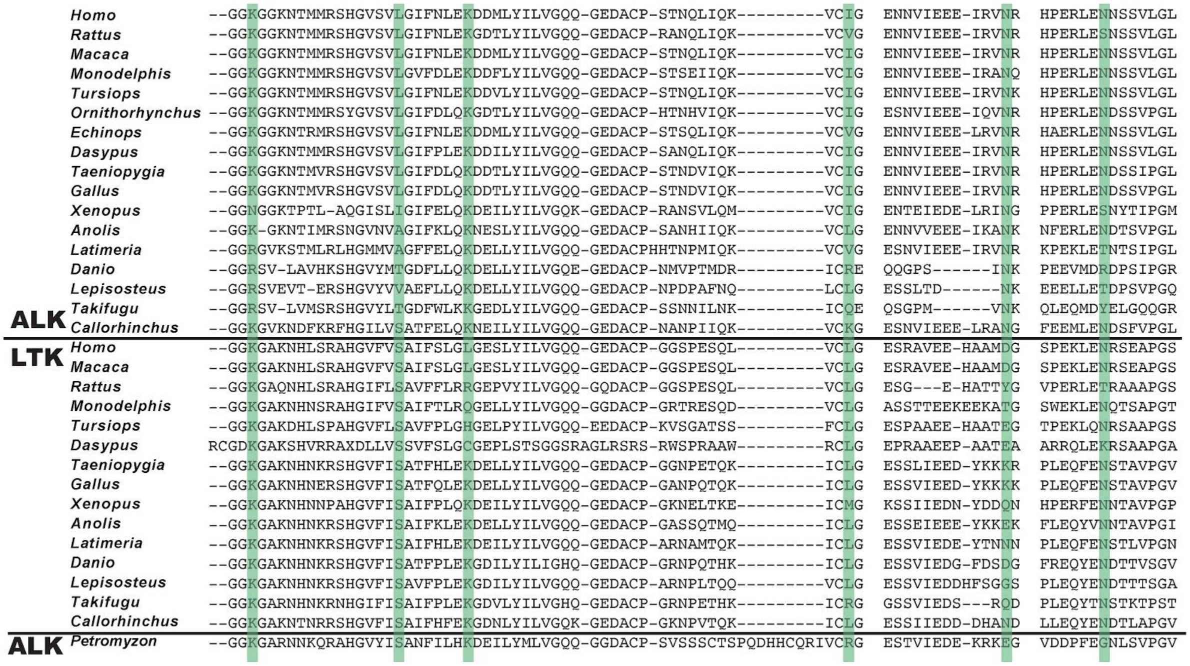

Figure S3. Analysis with DIVERGE (Gu et al. 2013) identified sites that exhibited significant substitution rate shifts among lineages between vertebrate ALKs and LTKs (green).

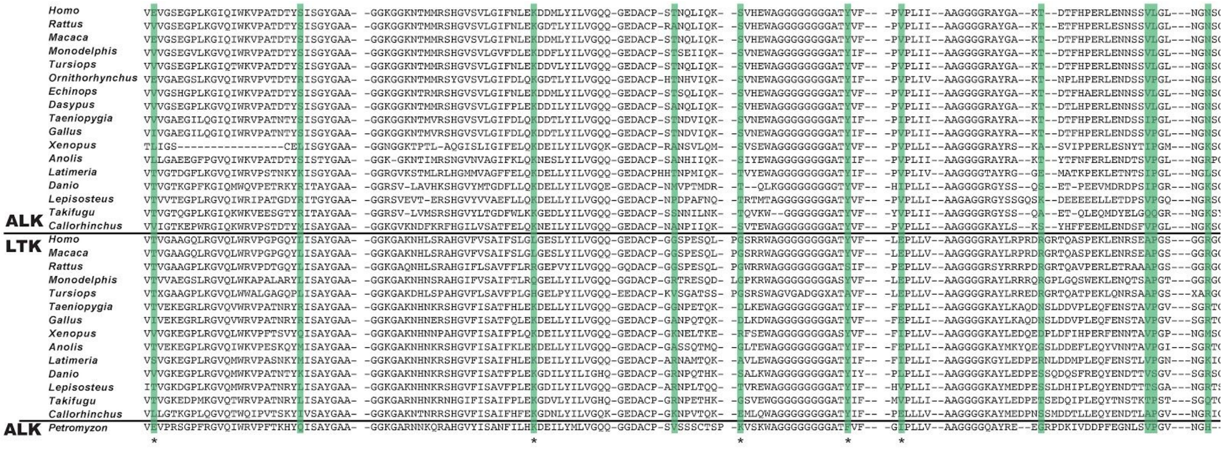

Figure S4. Analysis with `DIVERGE` (92) identified sites that exhibited significant substitution-rate shifts among lineages within vertebrate LTKs (green).

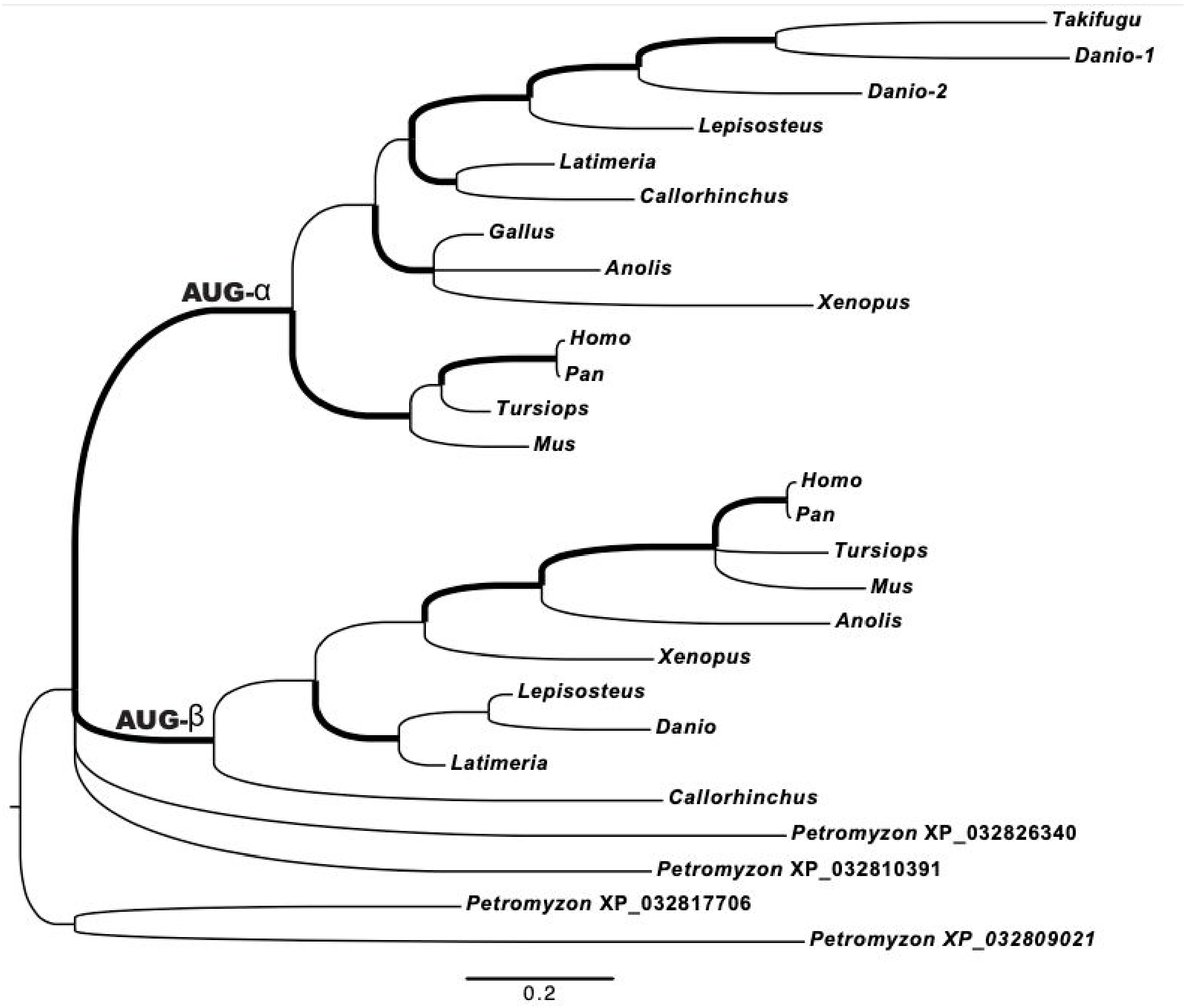

Figure S5. Phylogeny of vertebrate AUG, rooted with lamprey AUGs for display purposes. The phylogeny was inferred from a joint amino-acid alignment of AUG using `MrBayes` (93, 94). Branches with strong support (PP > 0.98) are in boldface.

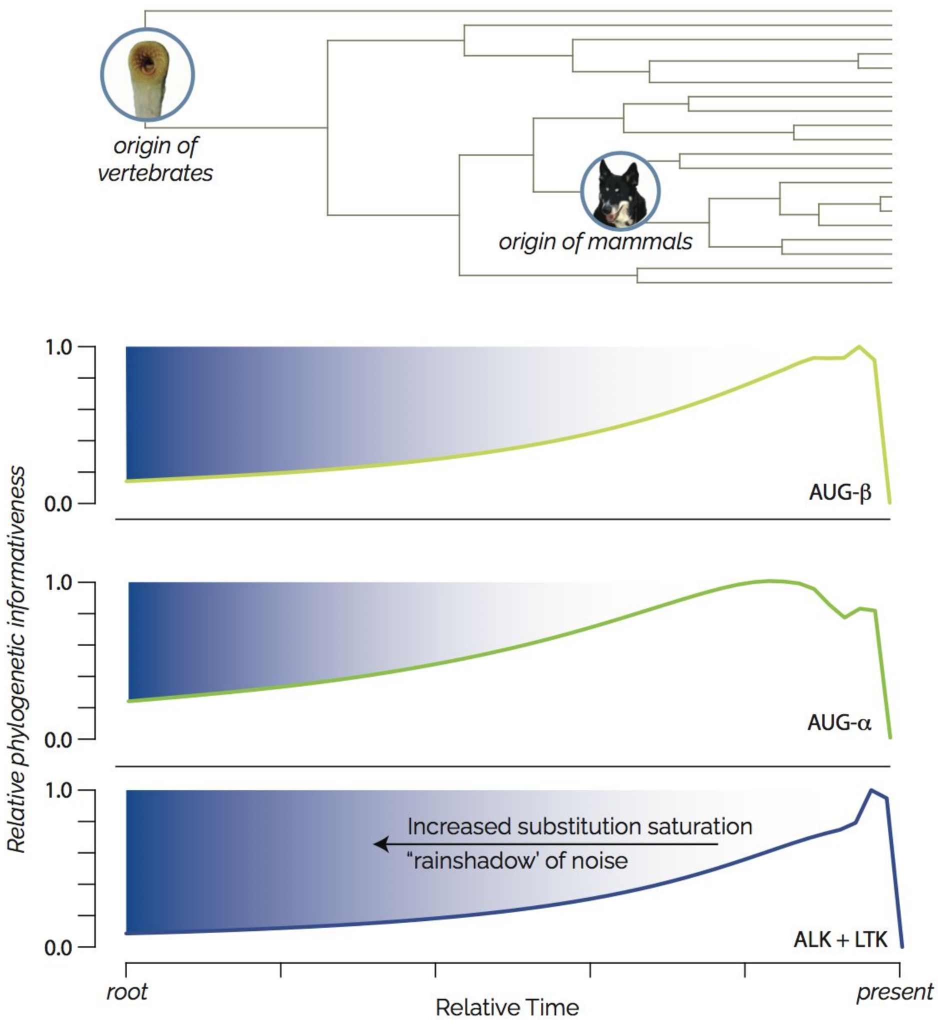

Figure S6. Phylogenetic informativeness profiles of AUG-α, AUG-β, and ALK and LTK homologs across vertebrate phylogeny (PhyInformR; 54). The decline in informativeness towards the origin of vertebrates indicates increased saturation of substitutions.

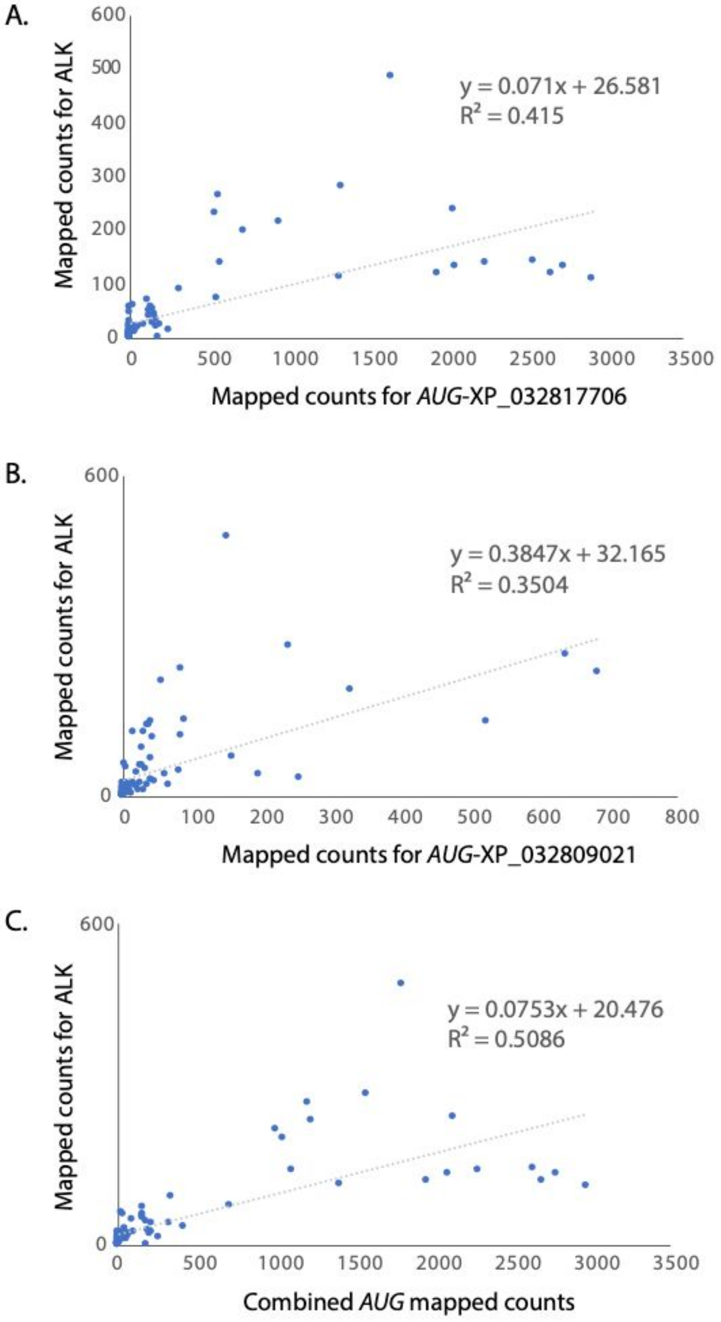

Figure S7. Correlation between RNAseq reads mapped to *ALK* and RNAseq reads mapped to the two expressed *AUG* genes in lamprey for A) *ALK* and *AUG*-XP_032817796; B) *ALK* and *AUG*-XP_032809021; C) *ALK* and both *AUG* genes combined. Reads are presented in Table S3.

Table S1. Homologs of ALK, LTK, JEB, HEN-1, and AUG identified in sampled invertebrate and vertebrate genomes.

Taxa	Common name	*ALK*	*LTK*	Ligand(s)
Ailuropoda melanoleuca	Giant panda	XP_019659786.1	XP_019658000.1	Aug-α (ENSAMEG00000009559)<br>Aug-β (ENSAMEG00000015012)
Amphimedon queenslandica	Sponge	XP_019848661?	Not found	Jeb-like (XP_019864501)
Anas platyrhynchos	Mallard	ENSAPLG00000013002	ENSAPLG00000011988	Aug-α (ENSAPLG00000012194)
Anolis carolinensis	Green Anole	ENSACAP00000008904	XP_008102570.1	Aug-α (ENSACAG00000005420)<br>Aug-β (XP_008114420.2)
Apis mellifera	Honey bee	XP_016766222	not found	Jeb (LOC100577600)
Aplysia californica	Sea hare	XP_012938087	not found	Jeb-like (XP_005090980)
Astyanax mexicanus	Blind cave fish	ENSAMXG00000013686	ENSAMXG00000011600	Aug-α1 (ENSAMXG00000014141)<br>Aug-α2 (ENSAMXG00000008347)
Bos taurus	Cow	ENSBTAG00000007379	XP_019823375	Aug-α (XP_010799138.1)<br>Aug-β (XP_010810318.1)
Branchiostoma floridae	Lancelet	XP_019615446 ?	not found	Jeb-like (XP_019621150)?
Caenorhabditis elegans	Nematode	SCD-2 (WBGene00004740)	not found	Hen-1 (WBGene00001841)
Callithrix jacchus	Common marmoset	ENSCJAG00000008763	ENSCJAG00000020723	Aug-α (ENSCJAG00000011653)<br>Aug-β (ENSCJAG00000014625)
Callorhinchus milii	Ghost shark	XP_007895095.1	XP_007887733.1	Aug-α (XP_007892194.1)<br>Aug-β (XP_007904100.1)
Canis familiaris	Dog	ENSCAFG00000005297	XP_022268223.1	Aug-β (XP_022268147.1)
Cavia porcellus	Guinea Pig	ENSCPOG00000008234	XP_023423243.1	Aug-α (XP_003464974.1)<br>Aug-β (ENSCPOG00000032897)
Chlorocebus sabaeus	Vervet Monkey	ENSCSAG00000011035	ENSCSAG00000007780	Aug-α (ENSCSAG00000006838)<br>Aug-β (ENSCSAG00000014724)
Choloepus hoffmanni	Hoffmann's two-toed Sloth	ENSCHOG00000012882	not found	Aug-α (ENSCHOG00000011961)<br>Aug-β (ENSCHOG00000008251)
Cimex lectularius	Bed bug	XP_014247913	not found	Jeb (LOC106666430)
Ciona intestinalis	Ciona	not found	not found	not found
Ciona savignyi	Ciona	not found	not found	not found
Danio rerio	Zebrafish	XP_691964.6	ENSDARP00000061546	Aug-α1 (ENSDARG00000091073)<br>Aug-α2 (ENSDARG00000042815)<br>Aug-β (ENSDARG00000074387)
Daphnia pulex	Water flea	EFX84662	not found	Jeb (DAPPUDRAFT_263759, _264276)
Dasypus novemcinctus	Nine-banded armadillo	XP_004452398.1	XP_012376273.1	Aug-α (ENSDNOG00000044907)<br>Aug-β (XP_004479910.1)
Dipodomys ordii	Ord's K=kangaroo rat	XP_012869014.1	XP_012865448.1	Aug-β (XM_013009307.1.1)
Drosophila melanogaster	Fruit fly	FBgn0040505	not found	Jeb (FBgn0086677)
Echinops telfairi	Lesser hedgehog tenrec	XP_012862293.1	not found	Aug-α (NP_001002919.2)
Eptatretus burgeri	Hagfish	not found	not found	not found
Equus caballus	Horse	XP_023474203.1	ENSECAG00000010591	Aug-α (ENSECAG00000009187)<br>Aug-β (ENSECAG00000011169)
Erinaceus europaeus	European hedgehog	XP_007523966.1	not found	Aug-α (XP_007529917.1)<br>Aug-β (XP_007516111.1)
Felis catus	Cat	XP_003984408.1	confirmed	Aug-α (XP_023107938.1)<br>Aug-β (XP_023104497.1)
Ficedula albicollis	Collared Flycatcher	ENSFALG00000011275	ENSFALG00000006086	Aug-α (ENSFALG00000002004)
Gadus morhua	Atlantic Cod	ENSGMOG00000014962	ENSGMOG00000011080	Aug-α ENSGMOG00000012987
Gallus gallus	Chicken	XP_015133623.1	XP_004941681.1	Aug-α (ENSGALG00000016364)
Gasterosteus aculeatus	Three-spined stickleback	ENSGACG00000012839	ENSGACG00000008800	Aug-α ENSGACG00000011449
Gorilla gorilla	Gorilla	ENSGGOG00000004378	ENSGGOG00000007006	Aug-α (ENSGGOG00000014328)<br>Aug-β (ENSGGOG00000002382)
Homo sapiens	Human	NP_004295.2	NP_002335.2	Aug-α (NP_001002919.2)<br>Aug-β (NP_997296.1)
Ictidomys tridecemlineatus	Thirteen-lined ground squirrel	ENSSTOG00000002811	ENSSTOG00000008387	Aug-α (ENSSTOG00000021139)<br>Aug-β (ENSSTOG00000003032)
Latimeria chalumnae	Coelacanth	XM_014487828.1	ENSLACP00000001197	Aug-α (XP_014350854.1)<br>Aug-β (XP_006000473.1)
Lepisosteus oculatus	Spotted gar	XP_015208377.1	XP_015194744.1	Aug-α (XP_015213224.1)<br>Aug-β (XP_015208959.1)
Loa loa	Eye worm	EJD76771	not found	Hen-1 (XP_003142890)
Loxodonta africana	African bush elephant	ENSLAFG00000014602	ENSLAFG00000017768	Aug-α (XP_023406155.1)<br>Aug-β (XP_010597850.1)
Macaca mulatta	Macaque	ENSMMUG00000010265	ENSMMUG00000008170	Aug-α (ENSMMUG00000017098)<br>Aug-β (ENSMMUG00000042459)
Macropus eugenii	Tammer wallaby	ENSMEUG00000000874	ENSMEUG00000002477	Aug-α (ENSMEUG00000002734)
Meleagris gallopavo	Turkey	ENSMGAG00000005339	XP_019471634.1	Aug-α (ENSMGAG00000013966)
Microcebus murinus	Mouse Lemur	ENSMICG00000000637	ENSMICG00000015875	Aug-α (XP_012638705.1)<br>Aug-β (XP_012645510.1)
Monodelphis domestica	Gray short-tailed Opossum	XP_007476091.1	XP_007480142.1	Aug-α (ENSMODG00000014263)<br>Aug-β (ENSMODG00000025613)
Mus musculus	House mouse	NP_031465.2	NP_976220.2	Aug-α (XP_017170402.1)<br>Aug-β (NP_001182661.1)
Mustela putorius furo	Ferret	ENSMPUG00000017981	XP_012910813.1	Aug-α (XP_004745767.1)

				Aug-β (XP_004777578.1)
Myotis lucifugus	Little brown bat	ENSMLUG00000010803	XP_023613374.1	Aug-α (ENSMLUG00000017708)
Nomascus leucogenys	Northern white-cheeked Gibbon	XP_012356733.1	XP_003266803.1	Aug-α (ENSNLEG00000008389)<br>Aug-β (ENSNLEG00000000759)
Ochotona princeps	Pika	ENSOPRG00000007284	ENSOPRG00000016526	Aug-α (XP_004582877.1)<br>Aug-β (XP_004580657.1)
Oreochromis niloticus	Tilapia	ENSONIG00000019537	ENSONIG00000020096	Aug-α1 (ENSONIG00000013462)<br>Aug-α2 (ENSONIG00000018754)<br>Aug-β (ENSONIG00000001740)
Ornithorhynchus anatinus	Platypus	XP_007665272.1	not found	Aug-α (XP_003429395.2)<br>Aug-β (ENSOANG00000021848)
Oryctolagus cuniculus	European rabbit	ENSOCUG00000001085	ENSOCUG00000026867	Aug-α (ENSOCUG00000029630)<br>Aug-β (ENSOCUG00000029695)
Oryzias latipes	Medaka	XP_023807229.1	ENSORLG00000015434	Aug-α (ENSORLG00000013880)<br>Aug-β (ENSORLG00000005531)
Otolemur garnettii	Northern greater galago	ENSOGAG00000014350	ENSOGAG00000012850	Aug-α (XP_012666040.1)
Ovis aries	Sheep	ENSOARG00000019617	ENSOARG00000020370	Aug-α (XP_014948423.1)<br>Aug-β (XP_014953145.1)
Pan troglodytes	Chimpanzee	ENSPTRG00000011796	ENSPTRG00000006948	Aug-α (ENSPTRG00000011606)<br>Aug-β (ENSPTRG00000024172)
Papio anubis	Olive baboon	ENSPANG00000015024	ENSPANG00000018808	Aug-α (ENSPANG00000015099)<br>Aug-β (ENSPANG00000015221)
Pediculus humanus	Body louse	XP_002432431	not found	Jeb (XP_002425611)
Pelodiscus sinensis	Chinese softshell turtle	ENSPSIG00000010494	ENSPSIG00000014814	Aug-α (ENSPSIG00000002370)<br>Aug-β (ENSPSIG00000014916)
Petromyzon marinus	Sea Lamprey	XP_032804141<br>XP_032818754	not found	Aug XP_032817706<br>Aug? XP_032826340<br>Aug? XP_032810391<br>Aug? XP_032809021
Poecilia formosa	Amazon molly	ENSPFOG00000011525	ENSPFOG00000005843	Aug-β (ENSPFOG00000021789),<br>Aug-α1(ENSPFOG00000001067)<br>Aug-α2 (ENSPFOG00000022096)
Pongo abelii	Orangutan	ENSPPYG00000012522	ENSPPYG00000006376	Aug-α (ENSPPYG00000012693)<br>Aug-β (ENSPPYG00000018583)
Procavia capensis	Rock hyrax	ENSPCAG00000008629	ENSPCAG00000005531	Aug-α (ENSPCAG00000012119),<br>Aug-β (ENSPCAG00000007706)
Pteropus vampyrus	Greater flying fox	ENSPVAG00000011977	ENSPVAG00000016352	Aug-α (ENSPVAG00000013389)<br>Aug-β (ENSPVAG00000005545)
Rattus norvegicus	Brown rat	ENSRNOG00000008683	ENSRNOG00000025130	Aug-α (ENSRNOG00000005154)<br>Aug-β (ENSRNOG00000048703)
Sarcophilus harrisii	Tasmanian devil	XP_023350365.1	ENSSHAG00000017193	Aug-α (ENSSHAG00000004800)<br>Aug-β (ENSSHAG00000009637)
Sorex araneus	Common shrew	XP_004620066.1	not found	Aug-α (XP_004616131.1),<br>(ENSSARG00000005846)
Strongylocentrotus purpuratus	Sea urchin	SPU_017036	not found	Jeb-like (SPU_018625)
Sus scrofa	Pig	ENSSSCG00000008533	ENSSSCG00000040414	Aug-α (ENSSSCG00000039057)<br>Aug-β (ENSSSCG00000033922)
Taeniopygia guttata	Zebra finch	XP_012427712.1	XP_002199013.2	Aug-α (ENSTGUG00000012951)<br>Aug-β (XP_004175323.1)
Takifugu rubripes	Fugu	ENSTRUP00000025266	XP_011616029.1	Aug-α (XP_011604626.1)
Tarsius syrichta	Philippine Tarsier	ENSTSYG00000009783	ENSTSYG00000027024	Aug-α (XP_008048005.1)<br>Aug-β (XP_008072903.1)
Tetraodon nigroviridis	Tetraodon	ENSTNIG00000008909	ENSTNIG00000017323	Aug-α (Q4RU86.1)
Tribolium castaneum	Red flour beetle	XP_008194070	not found	Jeb (TcasGA2_TC001096)
Tupaia belangeri	Northern tree shrew	ENSTBEG00000016366	ENSTBEG00000006236	not found
Tursiops truncatus	Atlantic bottlenose dolphin	XP_019798032.1	XP_019790652.1	Aug-α (XP_004327977.2)<br>Aug-β (XP_004325501.1)
Vicugna pacos	Alpaca	ENSVPAG00000004035	XP_015092567.1	Aug-α (XP_015106603.1)<br>Aug-β (ENSVPAG00000010782)
Xenopus tropicalis	Western clawed frog	XP_017949451.1	XP_002933782.2	Aug-α (XP_012818929.1)<br>Aug-β (XP_012820744.1)
Xiphophorus maculatus	Southern platyfish	ENSXMAG00000009140	ENSXMAG00000013305	Aug-α (ENSXMAG00000007504)<br>Aug-β (ENSXMAG00000018426)

Table S2. Gene-specific primers designed for RACE on lamprey *AUG*.

Identifier	Sequence[a]
**GSP #1**	GATTACGCCAAGCTTccatacgcgtgcacgcgctgctgg
**GSP #2**	GATTACGCCAAGCTTcgagatattcttccccatagcg
**GSP #3**	GATTACGCCAAGCTTccaataagagttccatgtacatgtcaccg
**GSP #4**	GATTACGCCAAGCTTgctgctgggacggagctgcgggtctc
**GSP #5**	GATTACGCCAAGCTTcgcattcccctcctccgaggacctcgc
**GSP #6**	GATTACGCCAAGCTTgcagttctaccggctctaccacaacacccgc g

[a] 15 nucleotides of anchor primer (upper case) and gene specific primer (lower case).

Table S3. Sea lamprey transcriptomic data from NCBI analyzed to evaluate expression of ALK and AUG like protein coding genes.

Experiments	XM_032961815	XM_032970449	XM_032954500	XM_032953130	XM_032962863	XM_032948250	BioProject	Size (MB)	Description
	XP_032817706	XP_032826340	XP_032810391	XP_032809021	XP_032818754	XP_032804141			
	AUG	*AUG*	*AUG*	*AUG*	*ALK*	Annotated *LTK*			
SRR2238818	112	153	0	42	70	9	PRJNA294488	21590	embryo stages 26–28
SRR3002835	2	0	0	12	2	0	PRJNA306044	537	1 day post-fertilization embryos
SRR3002837	5	0	0	67	19	0	PRJNA306044	3050	1 day post-fertilization embryos
SRR3002838	0	0	0	2	2	0	PRJNA306044	471	2 day post-fertilization embryos
SRR3002839	2	0	0	2	4	0	PRJNA306044	475	2 day post-fertilization embryos
SRR3002840	11	3	0	9	16	0	PRJNA306044	2445	2 day post-fertilization embryos
SRR3002841	2	0	0	9	18	0	PRJNA306044	502	2.5 day post-fertilization embryos
SRR3002842	2	2	0	4	10	0	PRJNA306044	506	2.5 day post-fertilization embryos
SRR3002843	1	1	0	29	55	0	PRJNA306044	2537	2.5 day post-fertilization embryos
SRR3002844	2	0	0	0	3	0	PRJNA306044	568	3 days post-fertilization embryos
SRR3002845	0	2	0	2	2	0	PRJNA306044	572	3 days post-fertilization embryos
SRR3002846	0	5	1	6	24	0	PRJNA306044	2943	3 days post-fertilization embryos
SRR3002847	0	0	0	8	5	0	PRJNA306044	573	4 days post-fertilization embryos
SRR3002848	0	0	0	7	8	0	PRJNA306044	577	4 days post-fertilization embryos

SRR3002849	3	0	0	41	28	0	PRJNA306044	2819	4 days post-fertilization embryos
SRR3002850	0	2	0	30	9	0	PRJNA306044	424	5 days post-fertilization embryos
SRR3002851	0	3	0	10	8	0	PRJNA306044	428	5 days post-fertilization embryos
SRR3002852	5	6	0	82	45	0	PRJNA306044	2198	5 days post-fertilization embryos
SRR369904	19	0	7	2	60	0	N/A	2533	meiotic testes
SRR388671	0	0	0	0	0	0	PRJNA50489	15	Parasitic Olfactory Epithelium
SRR388684	0	0	0	0	0	0	PRJNA50489	19	Adult Olfactory Epithelium
SRR388685	0	0	0	0	0	0	PRJNA50489	17	Adult Brain
SRR388686	0	3	0	0	0	0	PRJNA50489	16	Larval/Parasitic Brain
SRR388687	0	0	0	0	0	0	PRJNA50489	14	Larval Liver
SRR388688	0	0	0	0	0	0	PRJNA50489	13	Parasitic liver
SRR388689	0	0	0	0	22	0	PRJNA50489	1302	Larval intestine
SRR388690	1	0	0	3	1	0	PRJNA50489	1311	Larval Kidney
SRR388691	0	0	0	0	0	0	PRJNA50489	1368	Small Parasitic Kidney
SRR388692	0	1	0	0	4	0	PRJNA50489	1527	Embryo (Late Blastula, Stage 18)
SRR388693	1	0	0	1	4	0	PRJNA50489	1765	Embryo (Gastrula, Stage 20)
SRR388694	1	1	1	1	5	0	PRJNA50489	1849	Embryo (Neurula, Stage 22a)
SRR389301	185	0	0	0	0	0	PRJNA50489	1938	Adult Brain
SRR389302	0	0	0	0	0	0	PRJNA50489	1801	Parasitic Liver
SRR389303	0	0	0	5	9	0	PRJNA50489	1288	Small Parasitic Proximal Intestine
SRR389304	0	0	0	0	4	0	PRJNA50489	1206	Small Parasitic Distal Intestine
SRR389306	0	1	0	1	16	0	PRJNA50489	1240	Adult Intestine
SRR389307	1	0	0	1	0	0	PRJNA50489	1249	Adult Kidney

SRR389308	3	0	1	5	5	0	PRJNA50489	1909	Embryo (Neurula, Stage 22b)
SRR389309	0	0	0	6	3	0	PRJNA50489	1843	Embryo (Neural Crest Migration, Stage 23)
SRR389310	0	0	1	0	10	0	PRJNA50489	1832	Embryo (Neural Crest Migration, Stage 24c1)
SRR389311	2	3	0	0	11	0	PRJNA50489	1763	Embryo (Neural Crest Migration, Stage 24c2)
SRR5124568	148	0	0	47	25	0	PRJNA358429	3213	olfactory tissue after 24 hours with 5g/mL copper
SRR5124569	27	0	1	4	9	0	PRJNA358429	2940	olfactory tissue after 24 hours with 5g/mL copper
SRR5124570	188	5	0	25	23	1	PRJNA358429	2136	olfactory tissue after 24 hours with 30 g/mL copper
SRR5124571	167	4	0	254	33	0	PRJNA358429	3158	olfactory tissue after 24 hours with 10 g/mL copper
SRR5124572	132	3	1	26	54	0	PRJNA358429	2709	olfactory tissue after 24 hours with 30 g/mL copper
SRR5124573	155	3	0	21	41	0	PRJNA358429	2687	olfactory tissue after 24 hours with 0 g/mL copper
SRR5124574	52	2	3	17	19	0	PRJNA358429	2442	olfactory tissue after 24 hours with 5 g/mL copper
SRR5124575	38	1	2	6	20	0	PRJNA358429	2770	olfactory tissue after 24 hours with 5 g/mL copper
SRR5124576	151	0	1	6	52	0	PRJNA358429	3169	olfactory tissue after 24 hours with 0 g/mL copper
SRR5124577	91	18	0	14	21	0	PRJNA358429	2651	olfactory tissue after 24 hours with 0 g/mL copper

SRR5124578	128	7	0	197	40	0	PRJNA358429	2621	olfactory tissue after 24 hours with 30 g/mL copper
SRR5124579	158	6	0	60	39	0	PRJNA358429	2511	olfactory tissue after 24 hours with 30 g/mL copper
SRR5124580	49	4	0	20	16	0	PRJNA358429	2748	olfactory tissue after 24 hours with 10 g/mL copper
SRR5124581	31	0	0	22	8	0	PRJNA358429	2223	olfactory tissue after 24 hours with 10 g/mL copper
SRR5124582	125	3	0	34	49	0	PRJNA358429	3302	olfactory tissue after 24 hours with 0 g/mL copper
SRR5124583	168	12	1	35	20	0	PRJNA358429	2104	olfactory tissue after 24 hours with 10 g/mL copper
SRR5312374	1651	39	9	150	483	5	PRJNA377918	17894	spinal cord uninjured
SRR5312375	315	13	0	27	88	2	PRJNA377918	2433	spinal cord post injury 6 hours
SRR5312376	1945	13	0	16	118	5	PRJNA377918	5199	spinal cord post injury 1 day
SRR5312378	570	26	1	526	137	3	PRJNA377918	7179	spinal cord post injury 1 week
SRR5312379	544	60	0	685	230	2	PRJNA377918	10103	spinal cord post injury 2 week
SRR5312380	564	35	2	641	263	6	PRJNA377918	11737	spinal cord post injury 3 week
SRR5312381	716	42	0	328	196	2	PRJNA377918	8847	spinal cord post injury 4 week
SRR5312382	547	23	0	159	71	12	PRJNA377918	5257	spinal cord post injury 5 week
SRR5312383	2737	17	2	36	131	5	PRJNA377918	6554	spinal cord post injury 6 week
SRR5312384	948	22	0	57	214	8	PRJNA377918	12586	spinal cord post injury 12 week

SRR5312385	2045	47	10	84	238	4	PRJNA377918	14228	brain uninjured
SRR5312386	2661	32	1	30	117	5	PRJNA377918	7693	brain post injury 6 hours
SRR5312387	1337	33	1	239	280	1	PRJNA377918	6284	brain post injury 1 day
SRR5312388	2926	38	1	44	107	7	PRJNA377918	9861	brain post injury 3 days
SRR5312389	2054	30	1	37	131	1	PRJNA377918	7860	brain post injury 1 week
SRR5312390	1325	31	0	84	112	1	PRJNA377918	7615	brain post injury 2 week
SRR5312391	2244	32	2	42	138	4	PRJNA377918	9491	brain post injury 3 week
SRR5312392	2546	49	0	89	141	5	PRJNA377918	10065	brain post injury 4 week
SRR5312393	251	7	0	2	11	0	PRJNA377918	6349	brain post injury 5 week

Table S4. Selection analyses on mammal *ALK* and *LTK* using different models of PAML.
Table S4.1. Levels of selection (ω) within each clade, calculated under clade model C.

			Purifying selection class		Neutral site class		Divergent selection site class		ln L^a	P value[b] (df[c])
Clade	Tree length	κ^d	ω_0	p_0^e	ω_1	p_1^e	ω_2 ω_3 ω_4	p_2^e		
ALK	42.5	1.95	0.039	0.37	1	0.17	0.319 0.171	0.46	-89260.6	< 0.01 (1)
LTK	43.8	1.96	0.28	0.48	1	0.15	0.029 0.109	0.38	-89185.5	< 0.01 (1)
ALK + LTK	43.4	1.95	0.29	0.48	1	0.15	0.032 0.014 0.110	0.38	-89169.6	< 0.01 (2)
M_{2a_rel}	44.7	1.96	0.28	0.47	1	0.161	0.037	0.37	-89313.5	

[a] Natural log of the likelihood. [b] M_{2a_rel} was used as the null model for the likelihood ratio test, as in Weadick and Chang (2012). For the null model M_{2a_rel}, no foreground was defined. [c] degrees of freedom. [d] transition/transversion rate. [e] proportion of sites inferred to be in each selection category *i*.

Table S4.2. Levels of selection (ω) as determined by execution of branch models (PAML; 81).

Foreground branches[a]	Foreground ω_f	Background ω_b	ln L^b	P value
—	0.173		-92651.6	
ALK	0.152	0.181	-92643.9	< 0.01
LTK	0.259	0.162	-92619.1	< 0.01
ALK + LTK	0.153 0.258	0.167	-92617.2	< 0.01

[a] Nonsynonymous:synonymous substitution ratio ω was permitted to vary among branches in the phylogeny by designating the foreground branches and using the rest of the branches in the phylogeny as background. For the null model M_0 (first row), no foreground was defined. [b] Natural log of the likelihood.

Table S4.3 Levels of selection (ω) from models that allow the ratio ω to vary among sites.

Clade	Model[a]	ln L[b]	Parameter estimates[c]	Models Compared	P value[d]	Sites under selection[e]
ALK	M_0	-92651.6	$\omega_0 = 0.173$; tree length = 33.85; $\kappa = 1.79$			
	M_{1a}	-90488.6	$p_0 = 0.557$; $\omega_0 = 0.11$	M_{1a}:M_{2a}	1	901
	M_{2a}	-90488.6	$p_0 = 0.56$; $\omega_0 = 0.11$; $p_2 = 0.071$; $\omega_2 = 1.00$			
	M_7	-89118.9	$p = 0.61$; $q = 1.71$	M_7:M_8	< 0.01	901
	M_8	-89110.2	$p_0 = 0.99$; ($p_1 = 0.009$); $\omega = 2.84$			
LTK	M_0	-92651.6	$\omega_0 = 0.17$; tree length = 33.85; $\kappa = 1.79$			
	M_{1a}	-90488.6	$p_0 = 0.557$; $\omega_0 = 0.11$	M_{1a}:M_{2a}	1	901
	M_{2a}	-90488.6	$p_0 = 0.557$; $\omega_0 = 0.11$; $p_2 = 0.13$; $\omega_2 = 1.00$			
	M_7	-89118.9	$p = 0.61$; $q = 1.71$	M_7:M_8	< 0.01	
	M_8	-89110.2	$p_0 = 0.99$; ($p_1 = 0.009$); $\omega = 2.84$			

[a] M_0: one-ratio model; M_{1a}: neutral model; M_{2a}: selection model; M_7: Beta; M_8: Beta & ω.
[b] Natural log of the likelihood.
[c] Parameters p, p_0, p_1, p_2, and q govern the shapes of beta distributions within models.
[d] Likelihood-ratio tests were performed to test whether M_2 and M_8 models fit the data better than M_1 and M_7 respectively.
[e] Site(s) under significant positive selection (Bayesian posterior probability > 0.95) , identified via Bayes Empirical Bayes analysis (85).

Table S5. Pairwise evolutionary rates estimated for ALK and LTK for representatives of mammals, fishes, and other vertebrates.

Category	Species	ALK[a]	LTK[a]	Comparison of rates
Mammals	human vs opossum	0.103	0.244	ALK < LTK $P < 0.01$
	vervet monkey vs opossum	0.099	0.247	
	armadillo vs opossum	0.073	0.418	
	armadillo vs human	0.064	0.530	
	armadillo vs vervet monkey	0.063	0.533	
	mouse vs opossum	0.099	0.239	
	mouse vs human	0.073	0.273	
	mouse vs vervet monkey	0.066	0.272	
	mouse vs armadillo	0.057	0.450	
Fish	zebrafish vs ghost shark	0.120	0.172	ALK > LTK $P = 0.14$
	Latimeria vs ghost shark	0.186	0.150	
	Spotted gar vs ghost shark	0.317	0.139	
	Spotted gar vs *Latimeria*	0.186	0.125	
Others	*Anolis* vs zebra finch	0.173	0.163	ALK = LTK $P = 0.47$
	Xenopus vs zebra finch	0.145	0.155	
	Xenopus vs *Anolis*	0.146	0.172	

[a] dN/dS, calculated by the LPB93 method using PAML (95).
